# High genomic stability of Andes virus following successive passage *in vivo* in Syrian hamsters

**DOI:** 10.1128/jvi.00512-25

**Published:** 2025-07-24

**Authors:** Bryce M. Warner, Jérémie Prévost, Nikesh Tailor, Yvon Deschambault, Angela Sloan, Julina Allarie, Levi Klassen, Kathy Frost, Stephanie Booth, Jonathan Audet, Geoff Soule, David Safronetz

**Affiliations:** 1 Vaccine and Infectious Disease Organization, University of Saskatchewan 7235, Saskatoon, Canada; 2 Department of Biochemistry, Microbiology, and Immunology, University of Saskatchewan 7235, , Saskatoon, Canada; 3 Special Pathogens Program, National Microbiology Laboratory, Public Health Agency of Canada 85072 , Winnipeg, Canada; 4 Department of Medical Microbiology and Infectious Diseases, University of Manitoba 8664, Winnipeg, Canada; 5 Mycobacteriology, Vector-Borne and Prion Diseases Division, National Microbiology Laboratory, Public Health Agency of Canada 85072 , Winnipeg, Canada; University Medical Center Freiburg, Freiburg, Germany

**Keywords:** orthohantavirus, hantavirus, hantavirus pulmonary syndrome, Andes virus, disease modeling, virulence, pathogenesis

## Abstract

Andes virus (ANDV) is a South American hantavirus that causes hantavirus cardiopulmonary syndrome (HCPS), a severe respiratory infection with a case fatality rate as high as 40%. A critical model for studying HCPS is the Syrian hamster model, which faithfully recapitulates key aspects of the disease. Recent studies have shown that strains of ANDV from different origins, i.e., from rodent or human hosts, can produce widely different outcomes in the hamster model. CHI-7913, a human isolate, does not cause lethal disease and replicates poorly in hamster tissues compared with the classical rodent isolate Chile-9717869. Here, we passaged CHI-7913 *in vivo* in Syrian hamsters 25 times and infected groups of hamsters with virus from passages 2, 12, and 24 to assess whether viral adaptation occurs that might ultimately drive the phenotype of the virus during infection in this model. Very few mutations occurred during passage in hamsters, with only a single coding mutation discovered after 25 passages. Nearly all mutations occurring in hamsters occurred early, within two passages, and none of the passaged virus caused overt disease or pathology in infected hamsters, consistent with minimal changes in the viral genome. Early in infection, CHI-7913 viral RNA levels were still well below those seen in lethal Chile-9717869 infection. Overall, our data offer insights into the lack of selective pressure on hantaviruses in certain hosts and further evidence of distinct outcomes between rodent and human-derived viral isolates that should be studied further in additional viral species.

**IMPORTANCE:**

Infection of Syrian hamsters with Andes virus (ANDV) can result in disparate outcomes, depending on the source of the virus used for the infection. The ANDV strain CHI-7913 does not cause lethal disease in hamsters but is able to replicate in hamster tissues. We successively passaged CHI-7913 *in vivo* to study how continued infection influences adaptation in hamsters and whether passaging would lead to the development of a lethal model, as sometimes occurs with other viruses. Surprisingly, even after 25 passages, minimal mutations occurred in ANDV CHI-7913 genomes, with only one coding mutation present above consensus by passage 25, in the viral glycoprotein. Our data suggest that depending on both viral origin and the host, hantaviruses may face minimal selective pressure to mutate toward a disease phenotype. Studies with additional ANDV strains and other hantavirus species are warranted to further study this phenomenon.

## INTRODUCTION

The family *Hantaviridae* comprises 52 distinct viral species (ICTV 2023 release), including 35 within the *Orthohantavirus* genus, many of which are important human pathogens. Viruses within the family are enveloped, negative-sense RNA viruses that have a genome consisting of three segments, small (S), medium (M), and large (L), that encode three or four proteins (1). Several hantaviruses are zoonotic viruses that are carried by rodents, shrews, moles, and bats, though disease-causing species are carried primarily by murid and cricetid rodents. Human infection is caused by inhalation of aerosolized virus excreted or secreted from infectious rodents, or through direct contact (2). In the Americas, hantaviruses are the cause of hantavirus cardiopulmonary syndrome (HCPS), a severe respiratory disease with a case fatality rate as high as 40% depending on the causative virus and geographic location (2). There are no approved vaccines or therapeutics for the prevention or treatment of HCPS. Recently, concerns about reservoir hosts’ habitat destruction leading to more peridomestic rodent presence and the impact of climate change have been raised, with an anticipated increase in the incidence of hantavirus and other zoonotic virus infections (3).

Andes virus (ANDV; species *Orthohantavirus andesense*), carried by the long-tailed pygmy rice rat (*Oligoryzomys longicaudatus*), is one of the leading causes of HCPS in South America, mainly in Chile and Argentina (4). Infection with ANDV is the most well-studied of the HCPS-causing viruses due to a higher incidence rate and the occurrence of multiple small outbreaks of ANDV infection. In the 1990s, an outbreak of ANDV in Argentina occurred involving 16 cases that were linked epidemiologically, the first time human-to-human spread of a hantavirus was described (5). While limited person-to-person outbreaks have been reported, a large outbreak of ANDV occurred again in Argentina, in Epuyan, in 2018 and 2019. This outbreak spread to 34 individuals and caused 11 deaths, and was thought to have originated from a single individual infected via a rodent reservoir (6). Although ANDV remains the only hantavirus to show documented person-to-person transmission, the possibility remains for other hantavirus species. While ANDV transmission has been modeled in the classical Syrian hamster model of infection, due to the lack of a reverse genetics system, genetic signatures and specific residues that are able to drive human-to-human transmission have not been characterized. The molecular determinants of key aspects of hantavirus infection, such as pathogenesis and transmissibility, remain elusive.

There is a lack of suitable animal models to study hantavirus infection, and there are currently only two models in immunocompetent animals that faithfully recapitulate critical aspects of HCPS (7, 8). These include the macaque model of HCPS caused by Sin Nombre virus (SNV) and the often-used ANDV HCPS model in Syrian hamsters. The Syrian hamster model of HCPS has been well characterized and has been used to test the efficacy of various hantavirus vaccines and therapeutics, as well as study the pathogenesis of ANDV infection. Lethal HCPS in hamsters occurs following inoculation by several routes, including intraperitoneal, subcutaneous, intramuscular, intranasal, and intragastric (7, 9–12). In addition, ANDV HCPS has been reported for multiple ANDV strains. We previously reported that infection of Syrian hamsters with an ANDV strain isolated from a lethal human case, CHI-7913, does not cause lethal disease (13). Whereas CHI-7913 replicated in each of the tissues examined, replication was significantly lower compared to the rodent isolate Chile-9717869 that causes uniform lethality. Twenty-three amino acid differences were observed between the two viruses across the N, GPC, and L proteins. Without a reverse genetics system to study the impact of individual amino acids on infection outcome, we sought to identify residues that influence infection outcome in hamsters through serial passage of ANDV CHI-7913 *in vivo*. Here, we show that even after performing 25 serial passages in Syrian hamsters, ANDV CHI-7913 did not mutate significantly, acquiring only a single coding mutation. ANDV CHI-7913 also did not develop the ability to cause lethal disease; however, its capacity to replicate did change across passages. Most genetic changes occurred early on, suggesting limited evolutionary pressure toward lethality in hamsters.

## MATERIALS AND METHODS

### Viruses

ANDV strains Chile-9717869 (14) and the initial CHI-7913 stock used for passaging (15) were propagated using standard Vero E6 cell methodologies and titrated using a focus-forming assay as previously described (13) using rabbit hyper-immune serum as a primary antibody.

### Animals

Adult (>6 week old) male Syrian hamsters (*Mesocricetus auratus,* Charles River) were acclimated for a minimum of 1 week prior to experimental manipulations. Animals were given food and water *ad libitum* and monitored daily throughout the course of acclimation and experiments. Animals were group housed.

### Passaging

The initial group of hamsters (*n* = 4) was inoculated with 200 focus-forming units (FFU) of VeroE6-derived ANDV CHI-7913. At 8 days post-inoculation (DPI), two hamsters were euthanized, and the lungs were collected to use for subsequent passage inoculation. The remaining hamsters per passage were further monitored until 28 DPI for signs of disease. Immediately after collection, lungs were weighed, mechanically homogenized in standard cell culture medium (DMEM) with no additives, and clarified to remove debris. Inoculum for the next passage was 400 µL of a 10% wt/vol solution prepared in DMEM and injected in groups of four hamsters via the intraperitoneal route. Inoculum was monitored for the presence of ANDV using the molecular techniques outlined below. Passaging efforts continued for 25 rounds, with hamsters monitored daily for signs of disease.

### Serial sample study

To assess potential phenotypic differences arising from the passaging efforts, groups of 16 hamsters were inoculated intraperitoneally (IP) with a low (P2), middle (P12), and high (P24) passage inoculum and euthanized at defined times post-challenge to assess the kinetics of infection. For comparison, a group of hamsters was challenged with a lethal dose of ANDV strain Chile-9717869 (cell culture derived), and five animals were euthanized at the same time points to examine viral burden. Since infectious titers of virus could not be accurately determined in the passaged tissue homogenates of ANDV CHI-9713, all challenge inocula were standardized using reverse transcription-quantitative PCR (RT-qPCR) and, by comparison to a standard curve of ANDV Chile-9717869, were calculated to be 1 × 10^4^ FFU. On days 3, 7, and 12 post-inoculation, four hamsters per group (five for Chile-9717869) were euthanized and samples collected for virological, immunological, and histological analysis. The remaining four hamsters from the p2, p12, and p24 inoculation groups were euthanized at day 28 post-inoculation to assess viral persistence.

### Detection of ANDV RNA

Specimens (homogenates or tissues) were tested for the presence of ANDV using a previously described real-time RT-PCR assay targeting the nucleocapsid protein gene (13). Samples were extracted manually using Qiagen-based kits (passaging) or on a King Fisher Apex automated instrument (serial sample study) using Qiagen reagents.

For the detection of ANDV active replication using strand-specific RT-qPCR, we used a strand-specific 2-step RT-qPCR assay able to detect ANDV genome and antigenome as adapted from (16). RNA derived from ANDV challenge stocks was reverse transcribed by using the SuperScript IV First-Strand Synthesis System (Thermo Fisher Scientific), according to the manufacturer’s instructions. We used the ANDV-S130-F primer 5′-AAGGCAGTGGAGGTGGAC and ANDV-S291-R primer 5′-CCCTGTTGGATCAACTGGTT to detect the presence and relative amount of negative-strand and positive-strand ANDV S segment RNA, respectively. Two microliters of cDNA were removed for subsequent quantitative PCR (qPCR). Detection of ANDV S segment cDNA was tested by qPCR with the QuantiTect Probe PCR kit (Qiagen), according to the manufacturer’s instructions, using the following primers: ANDV-S130-F 5′-AAGGCAGTGGAGGTGGAC and ANDV-S291-R 5′-CCCTGTTGGATCAACTGGTT. The probe used was ANDV-S183-P, 5′-6FAM-ACGGGCAGCTGTGTCTACATTGGA.

### Serology

Serum samples were first inactivated by gamma-radiation (5 Mrad). To confirm seroconversion and evaluate antibody titers in ANDV-infected animals, an anti-ANDV N enzyme-linked immunosorbent assay was performed (13). Plates were coated with recombinant ANDV N protein produced using the standard baculovirus expression system. Serial twofold dilutions of serum samples (1:100 to 1:320,000) were used to determine anti-ANDV N IgG endpoint titers. HRP activity was quantified using TMB substrate (ThermoFisher), and optical density (OD) values were read at 650 nm. Positive samples were those that had an OD greater than the mean OD plus three standard deviations of the negative-control wells.

### Histology

Selected lung specimens from passaging and the serial sample study were formalin-fixed according to internal standard operating procedures. Post-fixation, samples were processed and stained as previously described (13).

### Blood counts and serum biochemistry

Serum biochemistry was analyzed using a VetScan VS2 analyzer (Abaxis Veterinary Diagnostics) per the manufacturer’s instructions. Blood counts were performed using a VetScan HM5 hematology system (Abaxis Veterinary Diagnostics) per the manufacturer’s instructions.

### Host immune responses

Serum samples used for cytokine analysis were first inactivated by gamma-radiation (5 Mrad). Cytokine responses in serum were analyzed using a 9-Plex Luminex Assay (Hamsters cytokine Panel-1, SKU H101-K) as per the manufacturer’s instructions. Samples were diluted 1:5, and plates were run on a Luminex MAGPIX instrument (Luminex, Austin, TX, USA). The analytes included IL-10, IL-2, IL-4, interleukin-6 (IL-6), interferon-γ (IFNγ), monocyte chemoattractant protein-1 (MCP-1), macrophage inflammatory protein-1α (MIP-1α), and vascular endothelial growth factor (VEGF).

### Viral sequencing

Selected passages were subjected to tiling amplicon sequencing. The primers used are shown in Table S1. The RNA was extracted using the Qiagen viral RNA kit. The reverse transcription was performed using SuperScript VILO (ThermoFisher) following the manufacturer’s recommendation, using 8 µL of RNA. The PCR step was carried out using Phusion U Multiplex Master Mix (ThermoFisher) with 2 µL of template in 25 µL reactions. The cycling conditions were 98°C for 2 minutes, 45 cycles of 98°C for 30 seconds, 65°C for 5 minutes, and a final extension at 72°C for 10 minutes. The PCR products were bead-purified using 0.8× MagMAX PureBind magnetic beads (ThermoFisher), washed twice with 80% ethanol, and eluted in 20 µL of 1 mM Tris pH 8.0 (ThermoFisher). The pools were quantified using a Qubit Broad Range dsDNA 1× kit (ThermoFisher) and combined at equal proportions where possible. The libraries were prepared using the Nextera XT library prep and sequenced on a MiniSeq (passages 5, 15, 20, 25) or a NextSeq2000 (passages 2, 4, 12, 24). The data were analyzed using the nf-ViralMutations pipeline (https://github.com/phac-nml/nf-ViralMutations) against reference sequences AY228237.1 (S segment), AY228238.1 (M segment), and AY228239.1 (L segment). The pipeline was set to remove non-primary alignments (using samtools) and clip primers (using bamclipper). Mutations were called where the depth was at least 20 reads and the frequency of the mutation was at least 5%. The data were plotted using R 4.4.2 using the tidyverse and patchwork packages.

### Statistical analysis

All results were analyzed and graphed using GraphPad Prism 8 software or R software. Statistical significance between groups was determined using a Kaplan-Meier analysis with log-rank test or one-way analysis of variance (ANOVA) where applicable.

## RESULTS

### Infection of Syrian hamsters with serial passages of ANDV CHI-7913

Many commonly used laboratory animal species are refractory to infection with certain wild-type viruses, as is the case for some coronaviruses, filoviruses, and arenaviruses (17–24). A common practice is to adapt natural virus isolates to individual species through serial infection until an isolate can cause infection or disease. This practice has been done previously with SNV, which is typically cleared by Syrian hamsters, producing an isolate after only five passages that can readily replicate but still does not cause lethal disease even after 20 passages (25). We performed 25 serial passages of ANDV CHI-7913 through Syrian hamsters via intraperitoneal inoculation of virus obtained from lung tissue during the peak of infection (8 DPI) during the previous passage. Each passage consisted of two naïve hamsters that were monitored daily for disease signs and euthanized based on the kinetics of replication of our previous studies using ANDV CHI-7913 (13). One animal died during serial passaging experiments, but we were unable to confirm that the death was due to ANDV infection. Nevertheless, tissues from this animal were used in subsequent inoculum preparations and passages, and also did not result in lethal infection. Otherwise, all animals included in serial passages did not develop disease and survived until 28 DPI.

**Fig 1:**
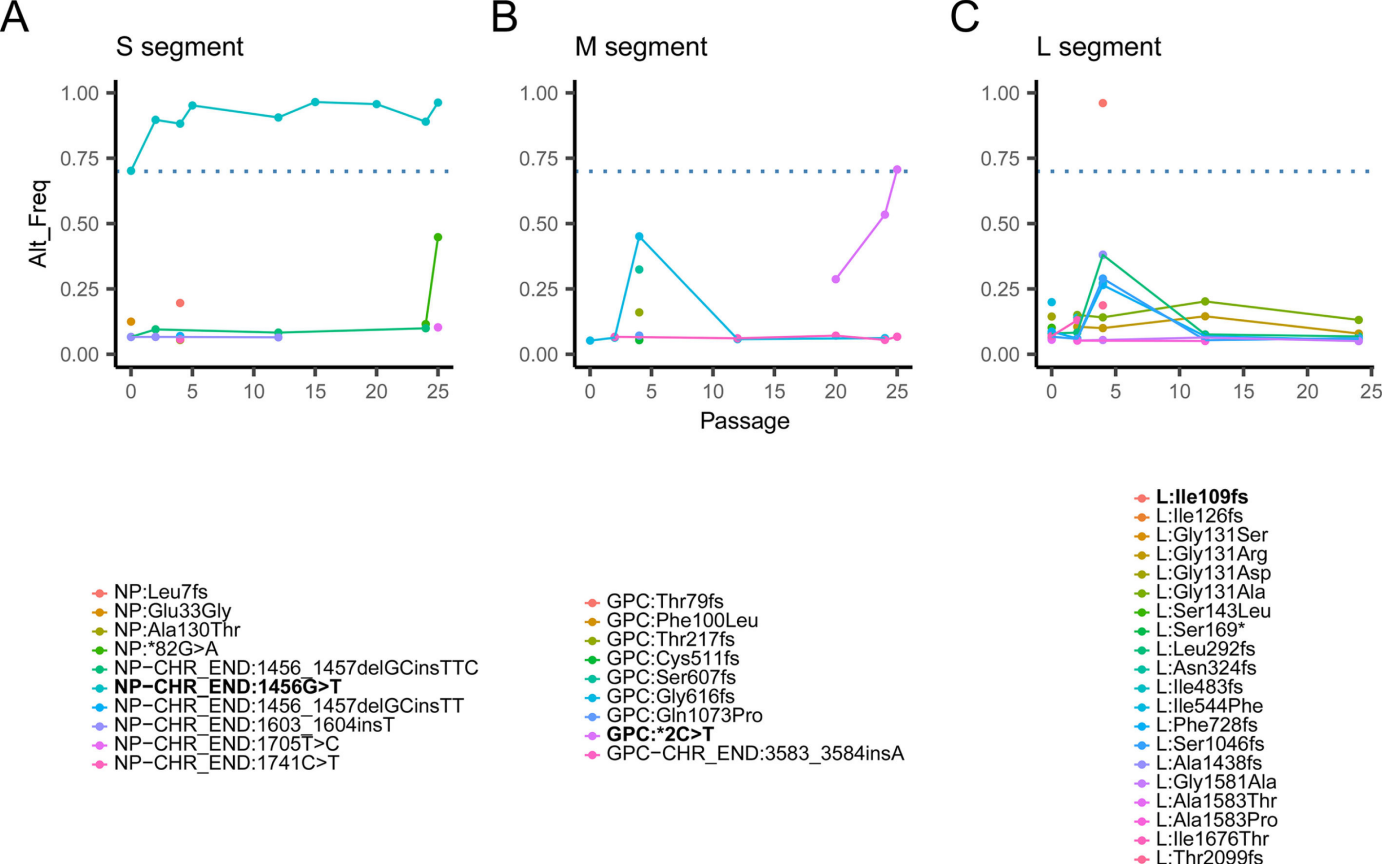
Frequency of non-synonymous and non-coding mutations in the ANDV CHI-7913 genome throughout the hamster adaptation passaging. Mutations for the ANDV CHI-7913 (A) S segment, (B) M segment, and (C) L segment are plotted across the passages. Solid lines represent mutations that reached consensus, and dashed lines represent mutations that did not.

**Fig 2:**
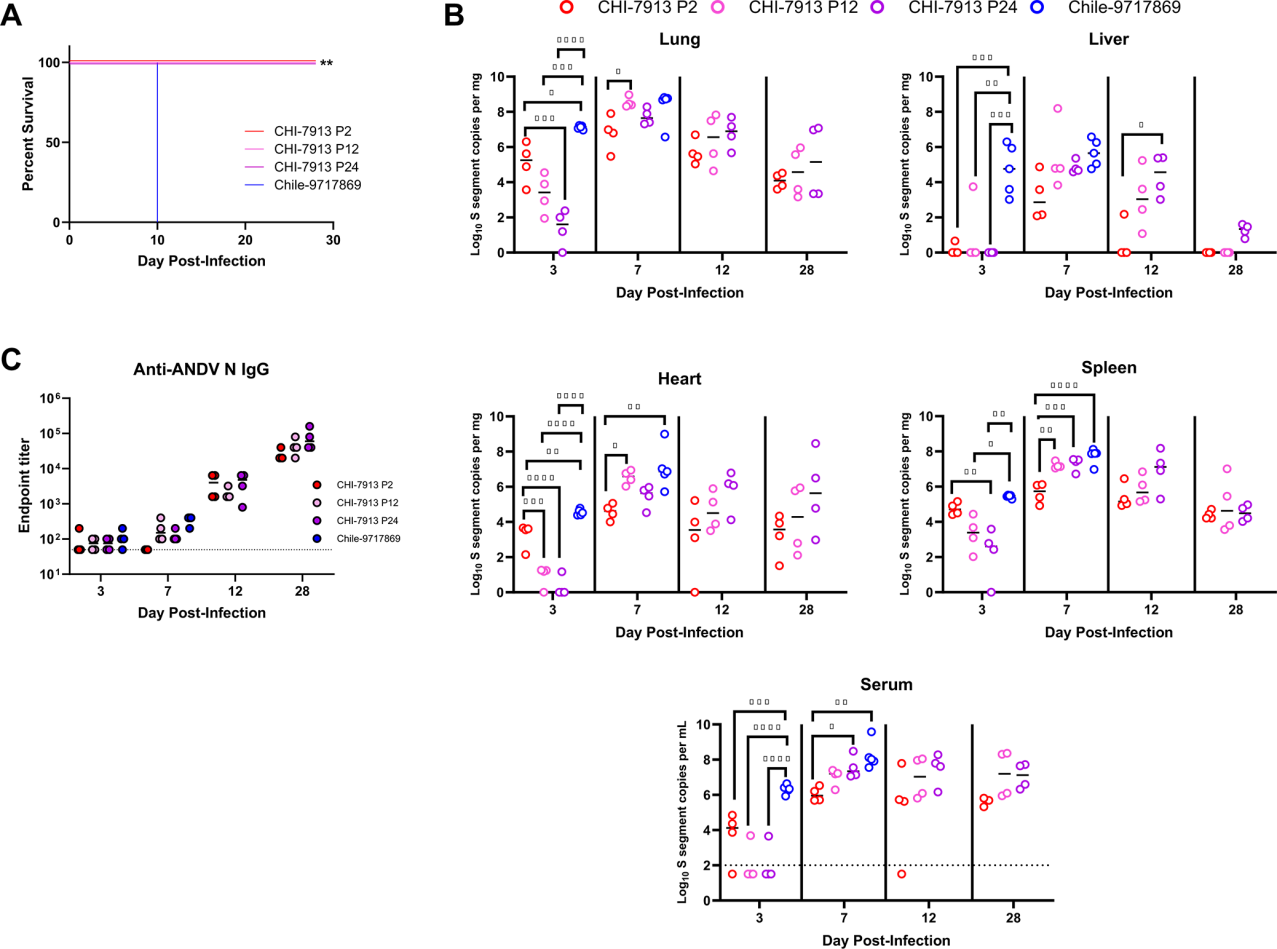
Survival and replication of Andes virus in Syrian hamsters. (**A**) Survival of hamsters infected with either ANDV strain Chile-9717869 or *in vivo* P2, P12, or P24 of ANDV CHI-7913. (**B**) Viral RNA levels in the tissues or serum of infected hamsters on 3, 7, and 12 DPI. (**C**) ANDV N-specific IgG endpoint titers in hamsters infected with ANDV strain Chile-9717869 or *in vivo* P2, P12, or P24 of ANDV CHI-7913. *n* = 16 hamsters per virus, with 4 euthanized on 3, 7, and 12 DPI and 4 kept for survival analysis (28 DPI). In B and C, medians and individual data points are shown. Statistical significance assessed by Log rank test in A, and by one-way ANOVA in B. *P < 0.05, **P < 0.01, ***P < 0.001, ****P < 0.0001.

Tiling amplicon sequencing was carried out on select passages of the virus. Sequencing coverage ranged from 93.5% to 99.4% at a minimum depth of 20 reads for all segments across all passages. Surprisingly, only one mutation made consensus on the S segment (G1456T; Fig. 1A, non-coding), one mutation rose to just above consensus level by passage 25 on the M segment (GPC:*2C > T [C3452T]; Fig. 1B), and one temporary consensus mutation was found at passage 4 (Ile109 frameshift; Fig. 1C). The raw sequencing data for passages 2, 4, 5, 12, 15, 20, 24, and 25 are available on the Sequence Read Archive (SRA) under BioProject PRJNA1256513. Even though only a single coding mutation was discovered, there is the possibility that changes to the viral genome that did not reach consensus, or that were seen in non-coding regions, could influence infection outcome or be selected for during infection and alter the outcome. To characterize any possible changes in infection outcome across early, middle, and late stages of passaging, we infected 16 hamsters each with ANDV CHI-7913 passages P2, P12, or P24, along with 12 animals infected with ANDV Chile-9717869 as a control for lethal infection and high levels of viral replication. Inocula of each challenge were standardized using qPCR and compared to a standard curve of ANDV Chile-9717869. Strand-specific RT-qPCR on each inoculum prep indicated that the genomic RNA (gRNA) to mRNA ratio was similar for each passage and Chile-9717869 (all within a ratio of 0.90–0.98). None of the animals infected with CHI-7913 from any passage succumbed to disease, whereas all four animals in the Chile-9717869 group kept for survival analysis had to be euthanized at 10 DPI after reaching humane endpoints (Fig. 2A). There were no obvious signs of disease in any of the CHI-7913 inoculated animals from any passage. Analysis of viral RNA levels in the lungs, liver, heart, spleen, and serum showed varying but present levels of viral RNA for all three passages during acute infection, despite no disease development (Fig. 2B). Interestingly, hamsters infected with P2 had significantly higher levels of viral RNA in the lungs and heart early after infection, at 3 DPI, before later passages had higher levels in the lungs, heart, spleen, and serum on day 7 (Fig. 2B). The only significant difference in RNA level seen at 12 DPI was in the liver between CHI-7913 P2 and P24 animals. Chile-9717869 RNA levels were consistently higher across all tissues and serum on 3 DPI, suggesting that efficient, early replication may drive pathology and disease, possibly from an exuberant inflammatory response. RNA levels on 7 DPI remained significantly higher in the Chile-9717869 group in the serum, spleen, and heart, whereas CHI-7913 passaged viruses reached similar levels in the lungs and liver. Because the remaining Chile-9717869 animals had to be euthanized at 10 DPI, we could only compare Chile-9717869 and CHI-7913 replication on days 3 and 7. All inoculated animals seroconverted by 12 DPI and had high anti-ANDV N IgG responses by 28 DPI (Fig. 2C). Despite high antibody titers, CHI-7913 RNA levels remained relatively stable between 12 and 28 DPI. Interestingly, at 28 DPI, animals infected with CHI-7913 still had detectable viral RNA in their lungs, heart, spleen, and serum (Fig. 2B), sometimes at high levels similar to what was seen during acute infection, suggesting viral persistence or delayed viral clearance.

**Fig 3:**
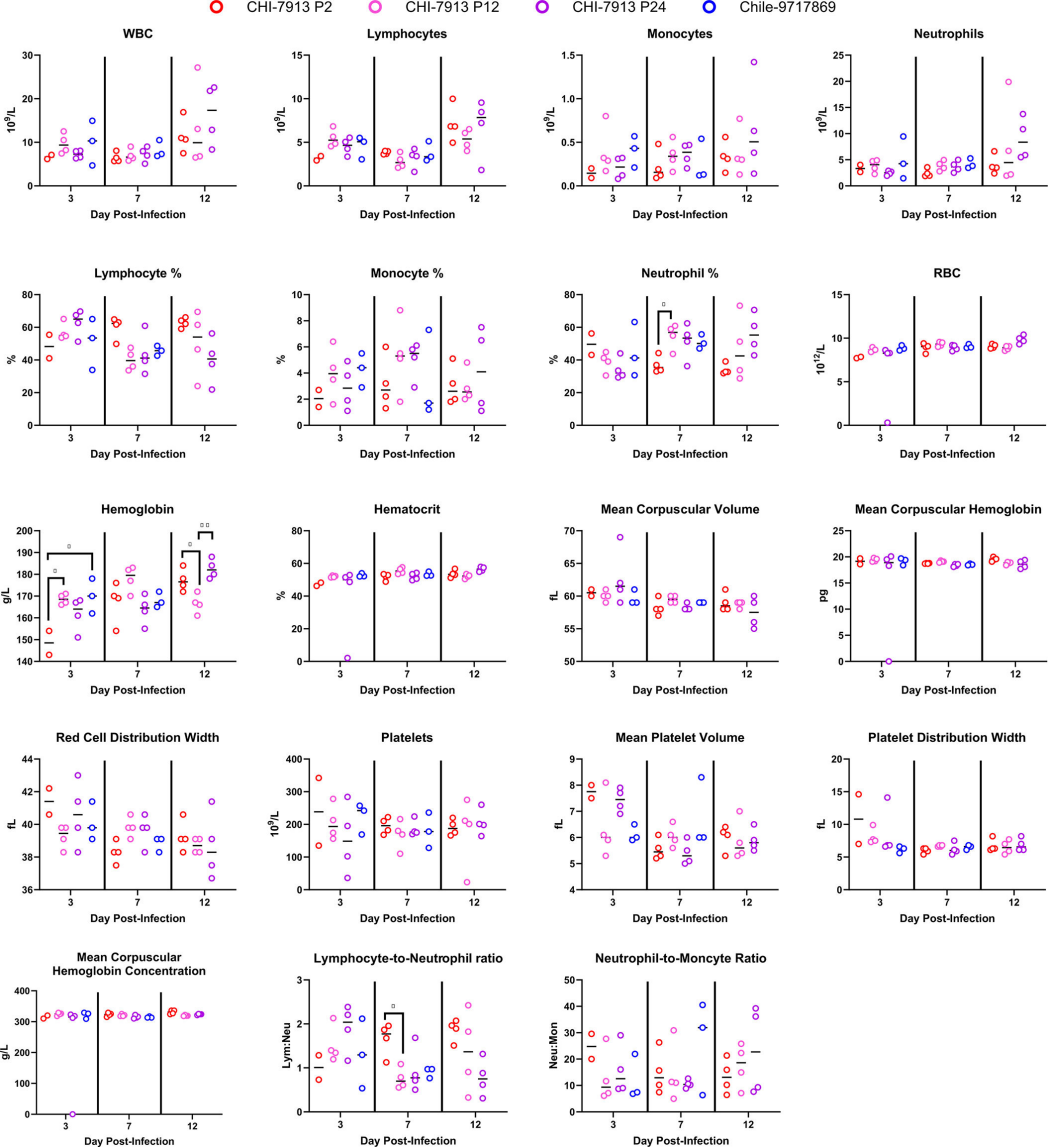
Hematological parameters in ANDV-infected hamsters. Hamsters were infected with either ANDV strain Chile-9717869 or *in vivo* P2, P12, or P24 of ANDV CHI-7913, and complete blood counts were performed with whole blood on 3, 7, and 12 DPI. Medians and individual data points are shown. Significance was assessed by one-way ANOVA. *P < 0.05, **P < 0.01.

**Fig 4:**
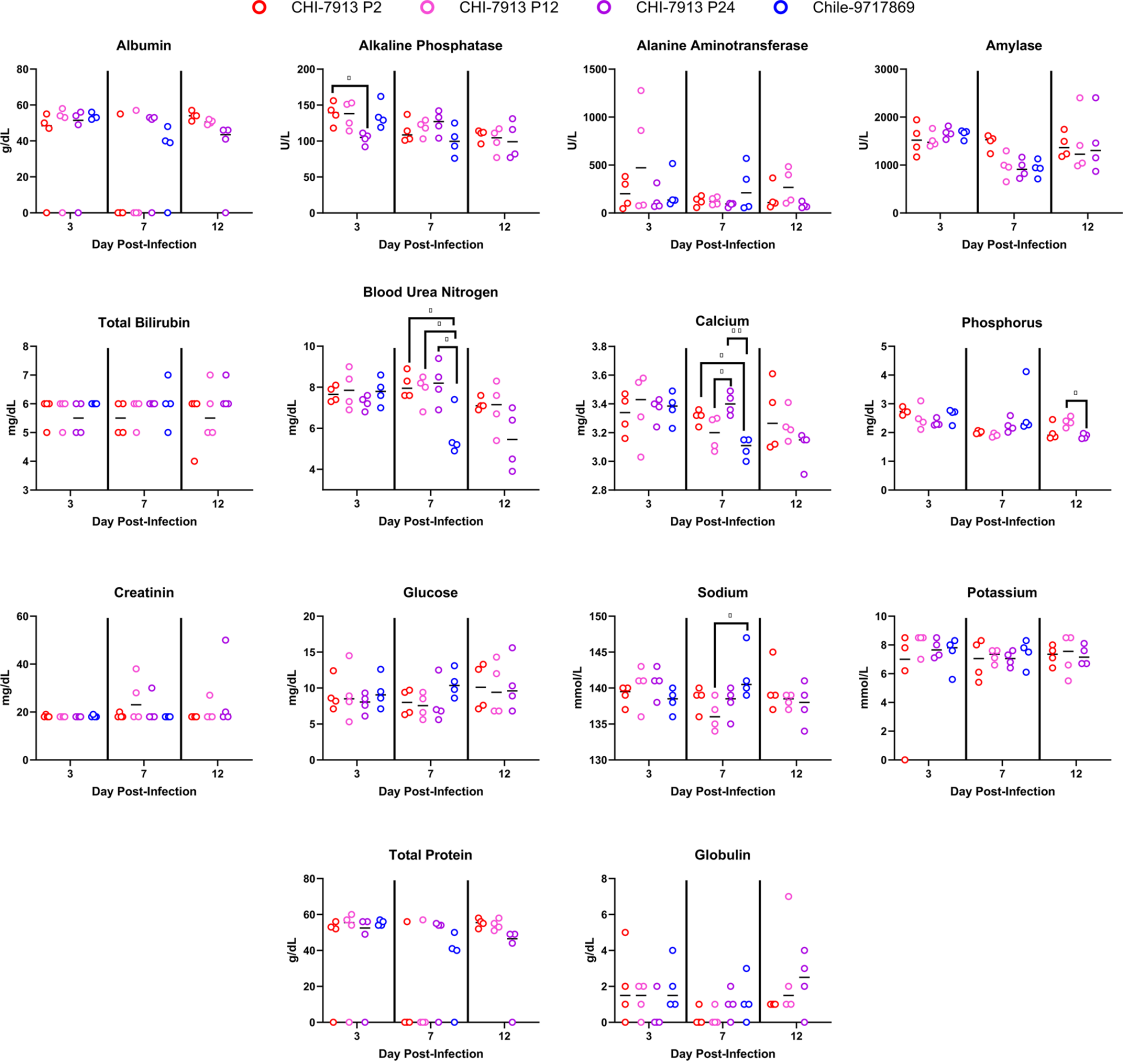
Serum biochemical parameters in ANDV-infected hamsters. Hamsters were infected with either ANDV strain Chile-9717869 or *in vivo* P2, P12, or P24 of ANDV CHI-7913, and serum biochemical analysis was performed with fresh serum on 3, 7, and 12 DPI. Medians and individual data points are shown. Significance was assessed by one-way ANOVA. *P < 0.05, **P < 0.01.

We performed complete blood count analysis and serum biochemistry analysis on animals infected with each CHI-7913 passage and Chile-9717869 and euthanized on days 3, 7, and 12 post-infection. Sufficient whole blood for hematological analysis was not collected from two animals in the CHI-7913 P2 group. For most hematological analytes examined, there were no differences between groups of animals infected with each passage or with Chile-9717869 (Fig. 3). Hemoglobin levels were significantly higher in Chile-9717869 and CHI-7913 P12 animals on day 3 compared with CHI-7913 P2. Hemoglobin was also lower in P12 animals compared to both P2 and P24 animals on day 12. Neutrophil percentage was higher in P12 animals than in P2 animals, but only on day 3, and neither P24 nor Chile-9717869 animals had significantly higher neutrophil percentage levels (Fig. 3). The only other significant difference between groups was in lymphocyte-to-neutrophil ratio, which was higher in P2 animals compared with P12 animals on day 7, but not higher than P24 or Chile-9717869 animals. Differences in certain serum biochemistry analytes were noted, including alkaline phosphatase (ALP), blood urea nitrogen, calcium, phosphorus, and sodium levels. ALP levels were reduced in P24 animals compared with P2 on day 3, but there were no other differences across groups (Fig. 4). Blood urea nitrogen levels were significantly lower in Chile-9717869 animals on day 7 compared with all CHI-7913 groups. Calcium levels were also significantly reduced in Chile-7917869 animals compared with each CHI-7913 group. The only other noted differences were decreased phosphorus levels in P24 animals on day 12 and increased sodium seen in Chile-9717869 hamsters compared with P12 hamsters on day 7 (Fig. 4). The minimal perturbations seen in hematological and biochemical measurements could be reflective of the delayed disease course seen in ANDV infection of hamsters infected with Chile-9717689 and the lack of or modest disease seen in CHI-7913 infected hamsters. Overall, our data indicate that serial passage of CHI-7913 does not lead to selection of a virus that results in disease, as measured by changes in hematological and biochemical parameters.

### Pathology

**Fig 5:**
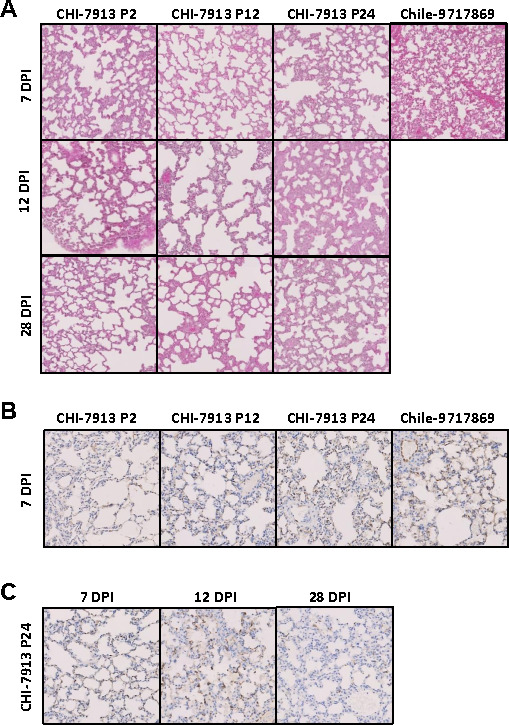
Histopathology in ANDV-infected hamsters. Hamsters were infected with ANDV CHI-7913 (P2, P12, P24) or Chile-9717869 via the intranasal route. (**A**) Hematoxylin and eosin staining was performed on the lungs from ANDV-infected hamsters taken at 7, 12, and 28 DPI (*n*  =  2/group). (**B and C**) Lungs from ANDV-infected hamsters taken at 7, 12, and 28 DPI were stained with rabbit hyperimmune serum and counterstained with hematoxylin (*n*  =  2/group). Representative sections are shown.

We collected tissues for histopathological analysis by hematoxylin and eosin, as well as immunohistochemical staining. Like previous findings, animals inoculated with CHI-7913 displayed little to no signs of microscopic pathology, which is consistent with the overall lack of clinical signs of respiratory disease (Fig. 5A). Although histological changes in lung samples from Chile-9717869-infected hamsters were minimal, this was likely an artifact of the timing of sample collection. Due to the rapidity of disease onset and ensuing death in these animals, terminal samples were not collected. As noted above, all hamsters infected with Chile-9717869 experienced terminal disease by day 10 and were euthanized due to severe respiratory distress. Immunohistochemical staining of ANDV antigens in lung specimens collected 7 days after inoculation revealed similar diffuse staining patterns in the mid- (P12) and late- (P24) passages of CHI-7913, which was comparable to that observed in Chile-9717869 infected hamsters (Fig. 5B). Time course analysis of the late passaged (P24) CHI-7913 demonstrated intense viral staining at days 7 and 12, which was largely diminished by day 28 (Fig. 5C). This pattern was similar to what was seen in P2 and P12 animals (data not shown) and suggests viral antigen clearance, despite the presence of viral RNA at this late time point.

### Immune responses

**Fig 6:**
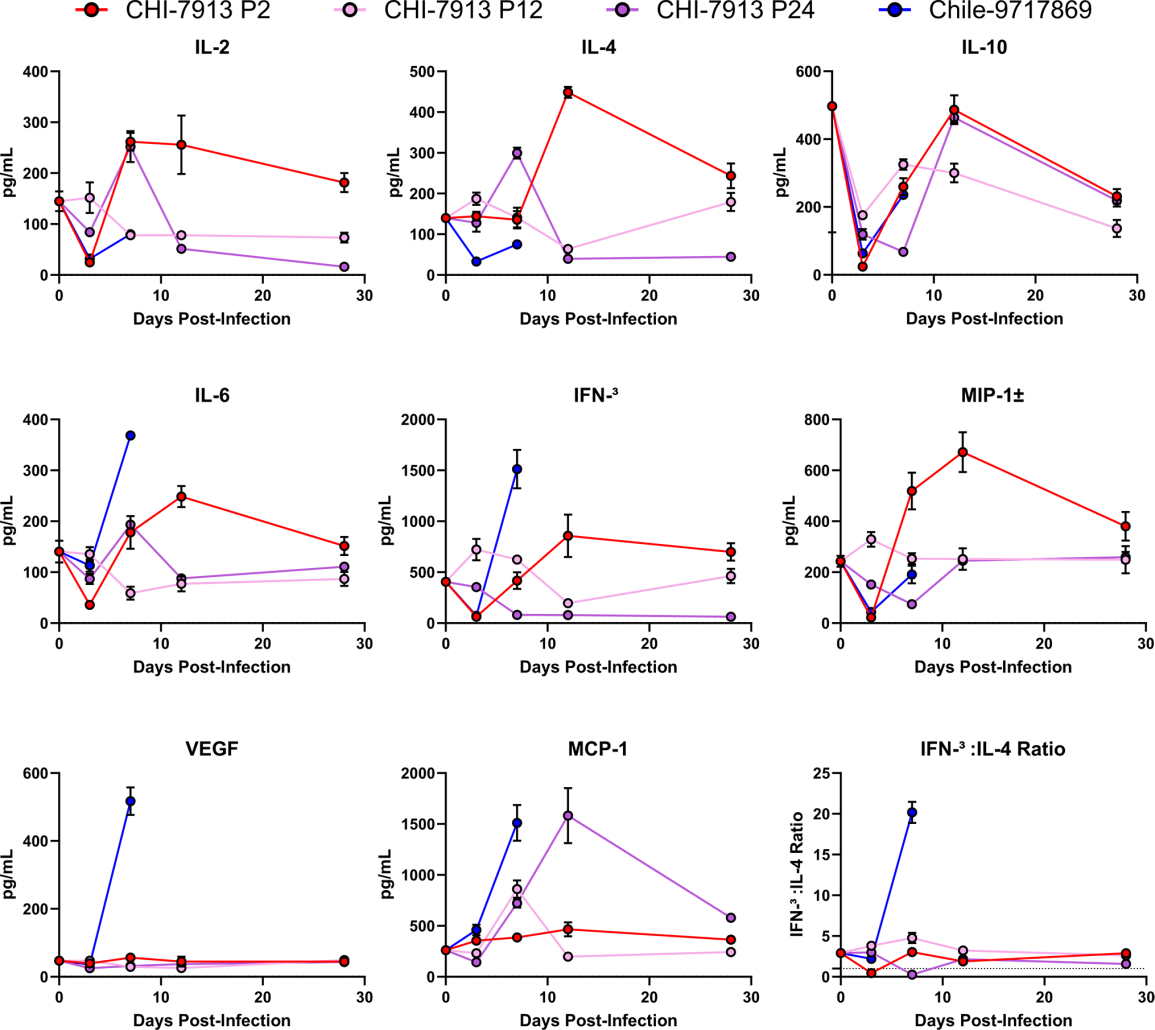
Cytokine production in ANDV-infected hamsters. Hamsters were infected with either ANDV strain Chile-9717869 or *in vivo* P2, P12, or P24 of ANDV CHI-7913, and cytokine levels in serum were assessed by Luminex assay. Mean + SEM are shown for each group at each time point. *n* = 4 per group per day post-infection.

CHI-7913 replicated efficiently in infected hamsters at each passage that was tested. To determine whether differences in host responses after infection drive disease, we examined the presence of different cytokines and serum biomarkers in infected hamster serum by Luminex. These included pro-inflammatory innate cytokines MCP-1, MIP-1α, and IL-6, T cell-produced cytokines IL-2, IL-4, IL-10, and IFN-γ, as well as VEGF, an angiogenic factor essential for vascular endothelial cells that has been shown to play a role in hantavirus pathogenesis (26–28). We tested the levels of each biomarker on days 3, 7, 12, and 28 post-infection to attempt to get a clear picture of the kinetics of the immune response. There were significant differences in several of the biomarker levels between groups on 3, 7, and 12 DPI (Fig. S1). Between CHI-7913 passages, there were distinct differences in biomarkers in P2 animals compared with P12 and P24 animals (Fig. 6). At 3 DPI, IL-2, IL-6, IL-10, IFN-γ, and MIP-1α levels were higher in P12 and P24 hamsters. At 7 DPI, several cytokines were increased in P2 animals, matching or surpassing levels seen in P12 or P24 animals. These included IL-2, IL-10, IL-6, IFN-γ, and MIP-1α. At 12 DPI, six out of eight biomarkers (IL-2, IL-4, IL-10, IL-6, IFN-γ, MIP-1α) were still significantly increased in animals infected with the P2 virus compared to levels seen in P12 or P24 animals. Only MCP-1 was increased in P24 animals relative to P2 and P12 animals on day 12. Aside from 12 DPI, there were as many differences between P2 and P12 as there were between P12 and P24, suggesting that despite a small number of changes or changes in only non-coding regions, some of these may play a role in alteration of host responses, immune evasion, or ultimately development of persistence.

There were clear differences in certain biomarker levels between CHI-7913 passage animals and Chile-9717869 animals. VEGF was significantly increased in Chile-9717869 hamsters on day 7 compared with all CHI-7913 groups, which did not show any increases compared to baseline (Fig. 6). MCP-1, IL-6, and IFN-γ were also increased significantly at 7 DPI in this group. Strikingly, the IFN-γ:IL-4 ratio, which is an indicator of a T helper 1-biased immune response and a strong adaptive inflammatory response, was much higher in Chile-9717869 animals compared with all CHI-7913 groups, which had either modest mixed IFN-γ:IL-4 ratios or strong Th2 biased ratios below 1 (Fig. 6). Overall, our data suggest that during lethal ANDV infection in hamsters, a strong initial inflammatory immune response, characterized here by increased IL-6 and MCP-1 production, followed by a robust Th1-biased inflammatory response, may drive pathology and lethal disease. 

## DISCUSSION

We passaged ANDV strain CHI-7913 serially *in vivo* in Syrian hamsters to attempt to drive a disease phenotype and to elucidate molecular determinants of ANDV pathogenesis in the hamster model. Our data demonstrate that despite repeated rounds of replication, in up to 25 passages in Syrian hamsters, ANDV acquires minimal changes to the genome and none that drive the viral phenotype toward a lethal infection. We compared infection with three different CHI-7913 passages to ANDV strain Chile-9717869, which offered clues to possibly important host responses causing lethality after Chile-9717869 infection, but not with CHI-7913. Viral RNA reached similar levels in CHI-7913 passage-infected hamsters compared with Chile-9717869, albeit with different replication kinetics, indicating that robust early replication may be critical for disease development. Serial passage resulted in more efficient replication in the spleen and serum in both P12 and P24 animals and in the lungs and heart of P12 animals compared with P2 at 7 DPI. Higher RNAemia may suggest a better capacity for the virus to spread systemically; however, this was not borne out in tissue RNA levels aside from those mentioned. We saw significant viral RNA levels maintained in hamsters at 28 DPI, suggesting persistent infection or delayed viral clearance. We did not carry animals beyond 28 days in our experiments; however, future studies examining whether ANDV CHI-7913 can persist for extended periods in hamsters could inform whether non-lethal strains are capable of long-lived or persistent infection, like what is seen in reservoir hosts. Histopathological analysis of infected tissues showed little to no pathology in the tissues of animals infected with CHI-7913 passages, despite high levels of viral RNA. Hantaviruses do not cause direct cytopathic effects, and cellular and tissue damage is typically the result of destructive hyper-inflammatory responses. This suggests that immune infiltration and immune-mediated damage are limited in CHI-7913 animals even with high viral replication. This may be due to differences in immune antagonism or because of differences in the early kinetics of viral replication. We only performed histopathological analysis on two animals per infection group, so there is a chance that we missed detectable differences in pathology or immunohistochemical staining between passages and with Chile-9717869. Our previous experience with this model with both viruses and the consistency seen in our pathological analysis across groups indicated that there were minimal differences, though we acknowledge that subtle differences may have been missed. We also performed a full hematological and serum biochemical work-up on infected animals in each group on the same days post-infection. Few of the clinical analytes measured showed significant differences between the groups, likely due to minimal disease in CHI-7913-infected animals and a relatively slow disease course in Chile-9717869-infected animals. Chile-9717869-infected animals typically show few clinical signs until around 1 day prior to succumbing to infection; therefore, it is possible that even on day 7, we missed significant perturbations in the parameters measured, and these would have been significantly altered on day 9 or 10, closer to lethality. Blood urea nitrogen levels being significantly elevated in Chile-9717869 animals suggests some kidney dysfunction during acute infection that was not seen in CHI-7913 animals, and which is consistent with hantavirus disease, even HCPS, which can have kidney involvement (29). We did not examine viral burden in the kidneys in this study; however, examination of the kidneys in the context of HCPS models could be worth investigating in the future, due to our better understanding of the overlap of the clinical presentations of HCPS and hemorrhagic fever with renal syndrome, caused by Old World hantaviruses.

Our results provide more data on how the origins of hantavirus isolates can impact infection outcome. Like what we have previously reported for SNV, serial passage of a hantavirus that is non-lethal in hamsters, in this case an ANDV strain, does not result in the development of a disease-causing or lethal phenotype (22). This contrasts with what is seen in other virus families for which mammalian species-adapted viruses have been generated, resulting in lethal strains. Serial passage of severe acute respiratory syndrome coronavirus (SARS-CoV) or SARS-CoV-2 can result in lethal mouse-adapted viruses (18, 20). Similarly, serial passage of natural isolates of filoviruses Ebola virus, Sudan virus, and Marburg virus can result in mouse- and guinea pig-adapted strains that cause lethal disease, and guinea pig adaptation of Lassa virus results in fully lethal strains, though these do not always recapitulate what is seen in human disease (17, 19, 21, 23, 24, 30). A critical difference between these viral families and hantaviruses may be the originating species, as both coronaviruses and filoviruses are thought to be mainly bat-borne, whereas pathogenic hantaviruses are rodent-borne; however, Lassa virus is also a rodent-borne Bunyavirus. Rodent-borne viruses may tend to adapt toward a non-virulent phenotype when passaged in a rodent species; however, the generalizability of the phenomenon needs to be tested experimentally. This could also be a general feature of hantaviruses, whereby selection toward a lethal phenotype outside their reservoir host poses a challenge. Indeed, work from our laboratory has reported that attempts to isolate SNV via inoculation of SNV-positive samples into deer mice, the reservoir host of the virus, did not yield infectious virus (31). SNV RNA-positive samples from human HCPS patients and SNV-infected non-human primates with HCPS inoculated into deer mice did not result in productive infection, whereas Vero cell-culture-adapted SNV was detectable at low levels in deer mice, suggesting diminished replicative ability. These results suggest that following spillover from deer mice into non-deer mouse hosts, SNV undergoes rapid evolution that prevents subsequent infection of deer mice, limiting chances of reverse zoonotic events. A similar type of adaptation has been reported for Puumala virus; however, this has not been previously seen with ANDV. However, since ANDV CHI-7913 was first isolated from a human patient, a similar adaptation phenomenon could be driving the reduced virulence in hamsters of CHI-7913.

The first report of lethal ANDV infection in hamsters used the strain Chile-9717869, which is a rodent isolate obtained from the reservoir host (9). Further work in Syrian hamsters showed that other ANDV strains also isolated from rodents, such as Chile R123 or ARG, could cause HCPS-like disease and provide additional lethal models to possibly study the breadth of anti-ANDV immunity and lethality of ANDV infection (14, 32). The complete sequence of CHI-7913 was reported in 2003 following isolation from the serum of an HCPS patient, a 10-year-old patient who caught the infection from his grandmother, and CHI-7913 has been used to study the ANDV sequence and protein structure (15). Our previous work showed that infection of Syrian hamsters with CHI-7913 does not result in lethal disease, a surprising finding (13). Between the often-used Chile-9717869 strain and CHI-7913, there were only 23 amino acid differences in the N, GPC, and L proteins; however, analysis of the NSs protein was not performed (13). Our studies indicated that there were differences in the host immune response following infection with these viruses; however, determining which positions in the genome are important for disease development could not be determined. Another study using the ANDV strain ARG examined lethality in hamsters using wild-type virus and multiple cell culture-passaged viruses (32). This study showed that successive passage in Vero E6 cells resulted in a loss of lethality, with 33% of animals surviving infection with the virus from passage 19. This study identified, in the ANDV strain ARG, eight sites of adaptation in ANDV coding regions after cell culture passage that resulted in attenuation in hamsters: three in N, one in the GPC, one in NSs, and three in L. This analysis also compared Chile-7917869, CHI-7913, and ARG strains, identifying sites in common between ARG and Chile-9717869 or ARG and CHI-7913, and identifying possible virulence markers. These included five sites in NSs, six sites in GPC, and 14 sites in L in Chile-9717869 that differ from wild-type CHI-7913 (13, 32). This study also revealed possible markers of human transmissibility in the ANDV genome by comparing ARG, CHI-7913, and sequences from several other ANDV isolates that have been associated with human-to-human transmission. One limitation of our study is that we were unable to compare directly the evolution, or lack thereof, of other ANDV strains, such as Chile-9717869 in hamsters or *in vitro,* to confirm that the lack of adaptation we saw in CHI-7913 is consistent across other isolates of ANDV. However, the polymerase is highly conserved, and the error rate is an inherent property of ANDV that is expected to be consistent across strains. Since we did not see significant changes arise in CHI-7913 genomes during our passaging, we were not able to identify mutations toward those previously seen in that comparison of ARG, CHI-7913, and Chile-9717869. Our data differ significantly in the fact that we saw minimal changes to the ANDV genome after successive passaging *in vivo,* which suggests minimal selective pressure on ANDV in hamsters. Interesting to note are the apparent differences in phenotype after infection with different CHI-7913 passages despite minimal changes to the genome. Reconciling this fact is difficult; however, changes to the virus in non-coding regions or minority variants that had amino acid changes that did not reach consensus could play a part in the differences seen in our experiments, since we did not sequence the virus from the animals in the serial necropsy study. There were several transient mutations that were present at varying levels, notably in the L segment that may have impacted viral replication, even if not reaching consensus (Fig. 1). Those changes were mostly detectable during early passages, prior to passage 5, and thus may have driven some of the differences seen in P2 animals specifically, compared with P12 and P24. These may also have impacted transcription and replication efficiency, as P2 RNA levels were higher than P12 and P24 early after infection. Although the dose of P2, P12, P24, and Chile-9717869 was normalized based on vRNA levels, our strand-specific RT-qPCR showed that the gRNA/mRNA ratio across all four inocula used was highly concordant, suggesting this was not a major contributing factor. Another possibility that could have impacted infection outcome is the presence of non-ANDV RNA or DNA, or host factors such as soluble immune mediators present in the lung homogenates that were used as inocula. Because these were not purified virus preps from cell culture, some of these present within the inocula could have influenced host innate responses and early viral replication. Other possible mechanisms underlying phenotypic differences, such as broader impacts on the host response or effects on host immune cell and infected cell epigenetics that alter viral entry, receptor expression, or immune gene expression, were unfortunately not examined in our study. However, these could be driving the differences seen between CHI-7913 passages, but likely do not significantly impact the lack of mutation seen in ANDV through successive passage, since these preparations were consistent throughout the study. A loss of lethality after passage in cell culture potentially indicates that rounds of infection in diverse hosts lead to a non-lethal phenotype, at least in hamsters. However, additional experiments examining this effect with Chile-9717869 would help shed more light on this phenomenon. A recent report also described infection of Syrian hamsters with the ARG-Epuyen strain of ANDV isolated during the Epuyen outbreak in 2018–2019 (33). This strain of ANDV, isolated from human cases, also did not cause disease in hamsters, whereas a classical ARG strain did cause disease, suggesting the difference in outcome with viruses from differing sources could apply to more ANDV isolates/strains (33). Without a larger number of human and rodent isolates to test in the hamster model, it will be difficult to test this hypothesis.

As previous studies compared viral nucleotide and amino acid sequences between ANDV strains, our previous comparison of Chile-9717869 and CHI-7913 showed differences in host responses following infection with these two strains (13). At the time of that study, our methodology was limited to examining transcript abundance for various cytokine genes, a method used previously in the ANDV hamster model (10). Here, we were able to utilize recently developed hamster-specific Luminex reagents to look at the presence of different serum biomarkers at the protein level. An advantage to this approach is that we can quantify expressed protein levels in serum, which may better correlate with the host response, as compared with mRNA transcript levels. A disadvantage is that we are only able to sample animal serum, rather than examining tissue-specific immune responses. Regardless, we used this approach to look at the kinetics of the immune response in passaged CHI-7913- and Chile-9717869-infected hamsters. Our data revealed significant differences in several cytokines throughout infection, including large differences in key markers between CHI-7913 and Chile-9717869 infected hamsters (Fig. 3). There was no clear progression toward an increased or decreased inflammatory phenotype after increased passage number, consistent with minimal changes in the viral genome. One of the critical features of ANDV infection is the innate immune antagonism of the NSs protein. NSs antagonize the type I interferon response through interaction with MAVS, reducing signaling leading to an antiviral state, allowing for increased initial viral replication (34). The Luminex panel used in our study did not include any type I interferon proteins to study possible differences in antagonism between CHI-7913 passages compared with Chile-9717869. Chile-9717869 RNA levels were significantly higher at 3 DPI, indicating that antagonism of this response may be more efficient in this strain; however, further differences in the impact of NSs mutations and NSs proteins from specific strains on type I interferon antagonism should be studied further. IL-6 has been shown to play an important role in driving inflammation and disease in other respiratory viral infections, such as SARS-CoV-2, and can be used as a marker for severe disease (35, 36). IL-6 was significantly increased in Chile-9717869 animals on day 7, while levels were lower in CHI-7913 animals. They were higher early after infection in P12 and P24 animals but were significantly higher in P2 animals by day 12. Higher levels of other cytokines in P2 animals on day 12 suggest a more prolonged inflammatory response after P2 infection, even though viral RNA levels were similar between all three passages on days 12 and 28. This indicates that the lack of viral clearance was not correlated with differences in the immune response at later time points. One of the more striking differences between ANDV strains was seen in the presence of serum VEGF. VEGF is an angiogenic growth factor that is important for angiogenesis during periods of hypoxia (37). It is often seen at high levels in asthma and diabetes, and the generation of new blood vessels can restore oxygen supply to tissues during times of hypoxic stress (37). VEGF is induced in cells that are not receiving adequate oxygen levels through the induction of hypoxia-inducible factor, a pathway that has been implicated in HCPS pathogenesis following infection of vascular endothelial cells and microvascular leakage and edema (27, 28). VEGF expression and VEGF receptor phosphorylation result in the degradation or downregulation of VE-cadherin, which promotes vascular leakage. Treatment of ANDV-infected hamsters with Vandetanib, a small molecule antagonist of the VEGF receptor, can prevent this phosphorylation and enhance tight junctions, with modest clinical effects *in vivo* in the hamster model (26). Our data suggest that the upregulation of VEGF following ANDV infection is a strong indicator of disease development and that ANDV or other hantavirus strains that can efficiently upregulate or induce VEGF production will cause disease. Further mechanistic experiments examining upstream events prior to VEGF production will be needed, since we did not see an increase in VEGF at 3 DPI; thus, hypoxic stress that triggers VEGF production is likely at a low level on day 3 or does not occur until after 3 DPI.

Interestingly, our results indicate that a strong Th1-biased immune response is correlated with lethal disease development following ANDV infection in hamsters. This is congruent with previous hypotheses that a strong inflammatory response is responsible for pathogenicity in HCPS (38, 39). Indeed, immunosuppressive treatments have been tested as a therapeutic for HCPS, though with limited data and mixed results (40). Prior studies examining the role of the immune response in hantavirus infection in hamsters showed that depletion of T cells in ANDV-infected hamsters does not influence disease outcome (41). Additionally, immunosuppression of Syrian hamsters using dexamethasone and cyclophosphamide results in lethal SNV infection, aiding in the development of a lethal SNV model in hamsters (42, 43). Depletion of alveolar macrophages also was shown not to prevent disease development in hamsters (44). These data suggest that neither virus-specific T cell responses nor an overexuberant inflammatory response are critical for disease development; however, our data might indicate that a Th-2 biased response, with fewer virus-specific CD8^+^ T cells and a more robust antibody response, may result in protective immunity. We did not see differences in the overall IgG response between groups of hamsters in our study; however, due to limited reagent availability, we are not able to examine ANDV-specific IgG isotypes, which may provide further information on the type of adaptive response generated. Higher IL-4 and IL-10 levels seen at later time points following infection are likely correlated with this type of Th2 bias. Further studies into the role of protective antibodies and T cell populations in HCPS disease are warranted. The development of a mouse model of infection to study the importance of these arms of the immune system on infection outcome would greatly advance the hantavirus field.

Here, we report that serial *in vivo* passage of the ANDV strain CHI-7913 in Syrian hamsters results in minimal changes to the genome of the virus. Up to 24 sequential passages of the virus did not result in a virus capable of causing lethal disease, and only resulted in a single coding mutation. Without the availability of a reverse genetics system to evaluate individual mutations in the ANDV genome, further studies using different ANDV strains as well as other New World hantaviruses will be critical in determining the impact of specific genomic sites on virulence and pathogenicity of hantaviruses, as well as studying the impact of virus origins and infected host on disease outcome. Our work also highlights the importance of obtaining hantavirus isolates from both humans and reservoir hosts and to sequence as many isolates as possible to study differences between circulating viruses and those that have spilled over. Given their possibly different phenotypes, they can provide clues and genomic signatures that might be important for human disease and viral ecology.

## Data Availability

Materials and data that are reasonably requested will be made available in a timely fashion. Sequencing data have been shared on the Sequence Read Archive (SRA) under BioProject PRJNA1256513.

## SUPPLEMENTAL MATERIAL

Figure S1 jvi.00512-25-s0001.pdf Comparison of cytokine levels in ANDV-infected hamsters.

SUPPLEMENTAL FILE 1 jvi.00512-25-s0002.docx Legend for Fig. S1.

Table S1 jvi.00512-25-s0003.pdf Primers used for viral sequencing.

ASM does not own the copyrights to Supplemental Material that may be linked to, or accessed through, an article. The authors have granted ASM a non-exclusive, world-wide license to publish the Supplemental Material files. Please contact the corresponding author directly for reuse.
