# Supplementary figures and images for: High genomic stability of Andes virus following successive passage in vivo in Syrian hamsters
Source: J Virol. 2025 Jul 24;99(8):e00512-25. doi: 10.1128/jvi.00512-25 (PMC12363185; doi:10.1128/jvi.00512-25)

● CHI-7913 P2    ● CHI-7913 P12    ● CHI-7913 P24    ● Chile-9717869

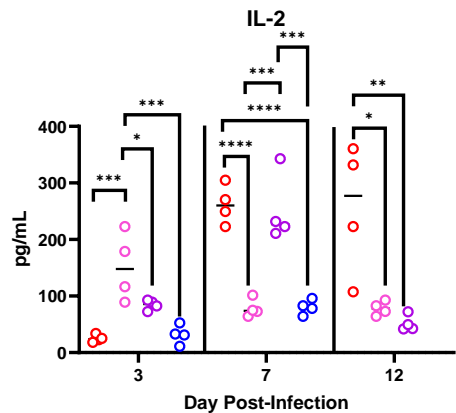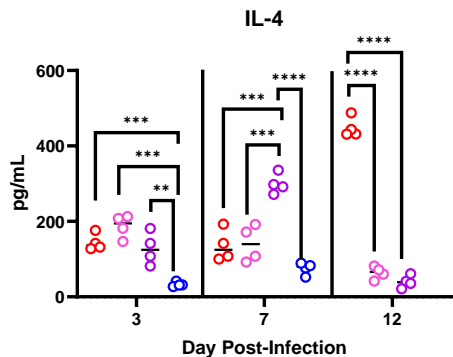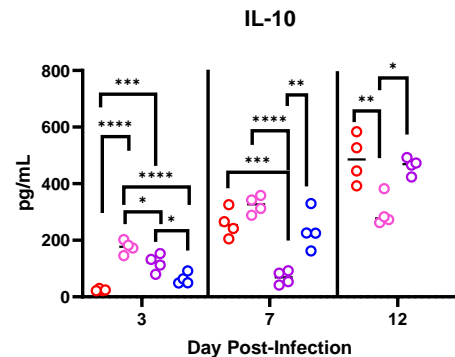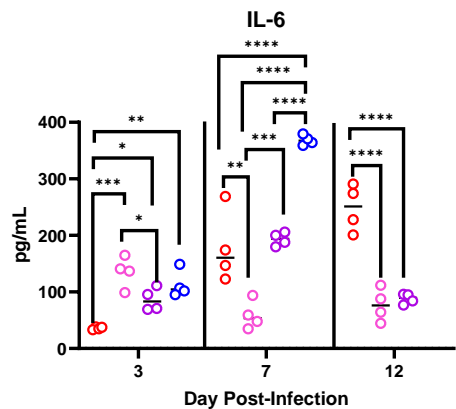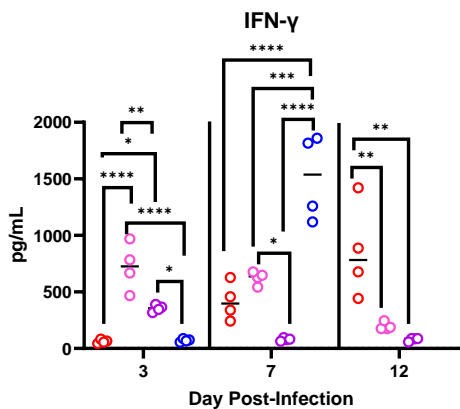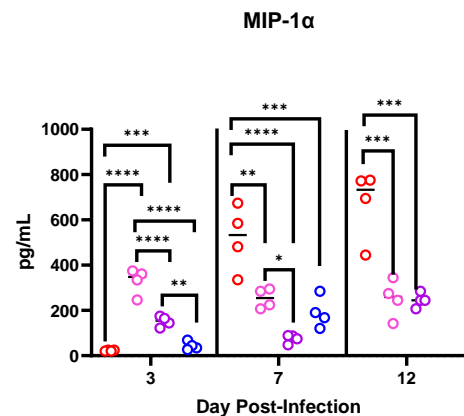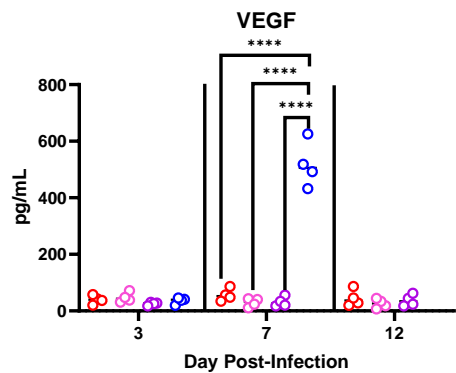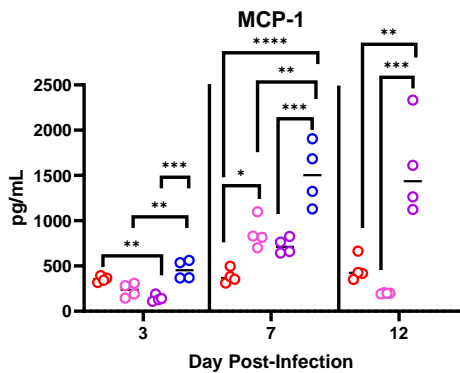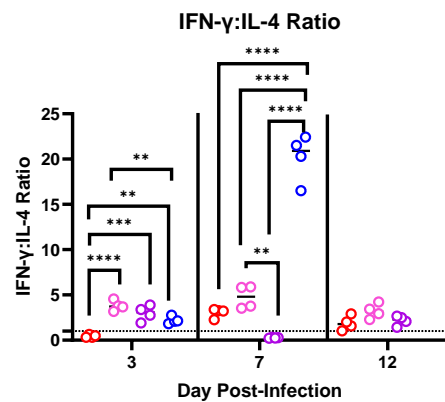

Supplement: Figure S1 — Comparison of cytokine levels in ANDV-infected hamsters. [file jvi.00512-25-s0001.pdf]
