# Supplementary material for: High genomic stability of Andes virus following successive passage in vivo in Syrian hamsters
Source: J Virol. 2025 Jul 24;99(8):e00512-25. doi: 10.1128/jvi.00512-25 (PMC12363185; doi:10.1128/jvi.00512-25)
Supplement: SUPPLEMENTAL FILE 1 — Legend for Fig. S1. [file jvi.00512-25-s0002.docx]

**Supplementary Figure 1. Comparison of cytokine levels in ANDV-infected hamsters.** Hamsters were infected with either ANDV strain Chile-9717869 or *in vivo* P2, P12, or P24 of ANDV CHI-7913 and cytokine levels in serum were assessed by Luminex assay. Cytokine levels in each group were compared by one-way ANOVA. Shown are medians and individual data points for each group. n = 4 per group per day. * = p < 0.05, ** = p < 0.01, *** = p < 0.001, **** = p < 0.0001.
