# Supplementary material for: High genomic stability of Andes virus following successive passage in vivo in Syrian hamsters
Source: J Virol. 2025 Jul 24;99(8):e00512-25. doi: 10.1128/jvi.00512-25 (PMC12363185; doi:10.1128/jvi.00512-25)
Supplement: Table S1 — Primers used for viral sequencing. [file jvi.00512-25-s0003.pdf]

Supplemental table 1. Primers used for viral sequencing.

Name	Segment	Sequence
S-1-1-F	S	CGAAAGCTGGAATGAGCACCCTC
S-1-1-R	S	CAAGCAATTGTCCTAAACCGTCCTG
S-1-2-F	S	AAAGCTCGAAATATAATAAGTCCTGTGATGG
S-1-2-R	S	GGGATTGAGCTAGCTGCCTTAGTTCAG
S-1-3-F	S	AAGGAGATCTCCAACCAAGAGCCAC
S-1-3-R	S	GAGGTAGTATGTGTTGAGGTAGAATAGGGGAG
S-2-1-F	S	AGAGAAATCATCTTTGAGATATGGGAATGTC
S-2-1-R	S	GGGGTGTTGCAATTTCTGTAAACAAGG
S-2-2-F	S	TTTGCATGTGCGCCTGACCG
S-2-2-R	S	CTTAACAACTATAATGTAGACTAACCCACCTCC
S-3-1-F	S	AAGAAAACATCACAGCACACGAACAAC
S-3-1-R	S	GCGTAATTTCCTCAGCCTTCATAGTAGATT
S-3-2-F	S	TCATGGGGTAAAGAAGCTGTGAATCA
S-3-2-R	S	GGATGAGGTAGTTATGTTGTAGGTTAGCAATG
M-1-1-F	M	GTGAGTTTAAAATGGAAGGGTGGTATCTG
M-1-1-R	M	GTGTGTGCTGGGTTGAAACATTGAC
M-1-2-F	M	ACTGACATTATCTCAACCTGCTCACACTTATG
M-1-2-R	M	GGGAACTAATGTTAAAGATACCATTTTCAGAATAG
M-1-3-F	M	AAGTCCAAAGATTTAGAGGATCCGAACAG
M-1-3-R	M	TGGCTTCTTAAGTGACTTTTTTAATGCCTC
M-1-4-F	M	AAAAGTAGATGTTATGTGGGTTTGGTATGG
M-1-4-R	M	TGCATGTTTGTTCTGTTCCTAACTGAATG
M-1-5-F	M	CAAATGATTGTTTAGTAACACCATCTGTGAAG
M-1-5-R	M	TTAATTGATGTCATAAAACTCGGGCACTC
M-1-6-F	M	GCATGTGATCTTGCTATGTGTTATGGAT
M-1-6-R	M	CCTCGGTAAGAGAATATGGAGTTATATATGAGTAAAG
M-2-1-F.2	M	ACAACTGATACCACAAATGCTGCATCC
M-2-1-R.2	M	TAGGGTGTACAAGCATCCTACTGACAACC
M-2-2-F	M	GAATAGTGGGACCTATCACAGCAAAAGTG
M-2-2-R	M	TCAAGACCCATCCGAAGCAGAATG
M-2-3-F	M	GTCTAAGGGTTCTGACATTTTCTTGTTCC
M-2-3-R	M	GAATTGACTCTTCCTTATTAGCTGGGTTTG
M-2-4-F	M	GTGATTCATGCTGAGATCCAACCC
M-2-4-R	M	AGAAATGGTATTACCTTGGTATTCGCAAAC
M-2-5-F	M	AAAGATTCATTCCAATCATTTAACTTAACAGAACC
M-2-5-R	M	ACTGAACATTATGATAGAGAGAATGAGTATCACAAC
M-3-1-F	M	GCTCTTGGAATCTGCTATACGCTGACAC
M-3-1-R	M	CAATCAGCTGACCTGTTACACAATGTGTC
M-3-2-F	M	CATGGGGTTCTGGTGTAGGGTTTAC
M-3-2-R	M	GTGAGGTTAGTTCCATAATAATCTTATAAAGTCAATAAC
L-1-1-F	L	TTAGAAAAATGGAAAAGTACAGAGAGATTCATC
L-1-1-R	L	GCTCTCTCATCTAACTTTATTAAATGCTCTCTGAC

Supplemental table 1 continued. Primers used for viral sequencing.

Name	Segment	Sequence
L-1-2-F	L	TTCATGTGTCAGGCCCTAAATTGAGATAC
L-1-2-R	L	CACATTTATGCTATCTATGACCTCACCAAATAAC
L-1-3-F	L	AATGCATGTGCAAAAATAATTGAGCAG
L-1-3-R	L	GTAAAAAGTGAAAAGAAAAGATTGACCTTAATG
L-1-4-F	L	AGATGAAACTATGTGCAATATTCGATAACCTTC
L-1-4-R	L	TGCTTATTCATGATATTAGCACATAAAACTTGAG
L-1-5-F	L	AACACGCAATATCAGTCTTAAGGGAATGTC
L-1-5-R	L	TCCATACTATCAATATATCGGTGTAATTTCCTAG
L-1-6-F	L	CAGTTGAAGACTTCTTGGCATTTTTTTC
L-1-6-R	L	TCTCTCAACTTTGCTTGTGCATAACACTATAG
L-1-7-F	L	TGTATGGTACTGCTGATGGAATGGTAAAC
L-1-7-R	L	CAGTAGGCTGGTAACTTGTGGCAAAAC
L-1-8-F	L	TGTAAGTTGTACATTCTCTAAAGAGTATGCTTGG
L-1-8-R	L	TCTTTGACATCCTGTTCATTTGCCTTAC
L-1-9-F	L	TTGATGGATTGGATAGAAAGGTTCAGAAG
L-1-9-R	L	GTACATTTATCATGAACTCTTTATTAATTGGCTG
L-1-10-F	L	CTTGTTGGCACATGCATATCATACATTG
L-1-10-R	L	CTGGCTCTAATACTCTTGTCACCCCAC
L-2-1-F	L	TGATATTATACAAGGCATGGAGAAGAAGATTATAC
L-2-1-R	L	ATTTCTCGGTTTCCGTGATTCATTG
L-2-2-F	L	GCATATCTGCCATCAACAATCAGTTCTG
L-2-2-R	L	GTGATATCTCTAACTAGATGACCTATATGCCATG
L-2-3-F	L	GCCGGTCTGCGTCGTTCTAAATAC
L-2-3-R	L	AATGATCAACTGTCAATCCTAACAAGCG
L-2-4-F	L	GGTCGGAGCAAGCGGTGTTTATC
L-2-4-R	L	TATCTGCTTCTGTTCTCTGGTATTTCCTGAC
L-2-5-F	L	ACAACATTACCAACTAGGGTGCGACTG
L-2-5-R	L	CAGACCTCCCGGAAAAGTAATGAGAC
L-2-6-F	L	TCATTCAGATGATGCATTATTTATTTATGGC
L-2-6-R	L	TCTCTACGAGTATGCTTATAGGATATGAATGC
L-2-7-F	L	AGATATGTATTGGGTTTATTTAAATTTTTGATGTC
L-2-7-R	L	TCACAGAATTTATTGGATTTTGTATAGCCTG
L-2-8-F	L	TATAAGTATGCTCTCACAGTAGATGAAGTCAGTG
L-2-8-R	L	TGAATAGGCTCTTCGGTTTGCTTG
L-2-9-F	L	CATACTGTGTGCAAACATATAACGAGGAAAC
L-2-9-R	L	TATTAGAGAATATGCAGCAGGAGGTATAGCAG
L-2-10-F	L	TCTGTGGATTTGTTTATGTTCAATGAACTATTAG
L-2-10-R	L	AGAATGTTGATACAGGGTCACGCG
L-3-1-F	L	GCACCTGGAACGGTATCAGCATTAG
L-3-1-R	L	TGCTGTGGTCTATAATCAGGGATGTTGAAG
L-3-2-F	L	ATGGGATTGAAGAAACATCTGAAGGTTATAG
L-3-2-R	L	CTTCGTAGCAAGACATCAACATGCC
